# Incidence of strabismus, strabismus surgeries, and other vision conditions in Prader-Willi syndrome: data from the Global Prader-Willi Syndrome Registry

**DOI:** 10.1186/s12886-021-02057-4

**Published:** 2021-08-12

**Authors:** Jessica E. Bohonowych, Caroline J. Vrana-Diaz, Jennifer L. Miller, Shawn E. McCandless, Theresa V. Strong

**Affiliations:** 1 Foundation for Prader-Willi Research, Walnut, CA USA; 2 University of Florida, Gainesville, FL USA; 3 University of Colorado Anschutz Medical Campus, Aurora, CO USA

**Keywords:** Prader-Willi syndrome, Strabismus, Vision, Registry

## Abstract

**Background:**

There is a relative lack of information on the incidence and treatment of vision problems in Prader-Willi syndrome (PWS). Using data from the Global PWS Registry, we performed a cross-sectional study of vision problems in PWS.

**Methods:**

Data, reported by caregivers who completed the Vision Survey in the Global PWS Registry between May of 2015 and March of 2020, were analyzed using descriptive statistics.

**Results:**

There were 908 participants in this survey, with a mean age of 14.5 years (range 0–62 years). The prevalence of strabismus in this population was 40 %, with no statistically significant difference in prevalence by genetic subtype. Ninety-one percent of participants with strabismus were diagnosed before 5 years of age. Of those with strabismus, 42 % went on to have strabismus surgery, with 86 % of those having their first strabismus surgery before 5 years of age and 10.1 % having more than one strabismus surgery. Additional vision issues reported included myopia (41 %), hyperopia (25 %), astigmatism (25 %), and amblyopia (16 %).

**Conclusions:**

The prevalence of strabismus, amblyopia, and hyperopia are considerably higher in the PWS population represented in the Global PWS Registry as compared to the general population. People with PWS should be screened early and regularly for vision problems.

## Background

Prader-Willi syndrome (PWS) is a rare genetic disorder with an estimated birth prevalence of 1 in 15,000 individuals, affecting males and females equally, as well as all races and ethnicities [1–3]. PWS is caused by the loss of expression of paternally-expressed genes on chromosome 15q11.2-q13, with ~ 60 % of cases due to a deletion of the paternal allele in this region (del), ~ 35 % of cases resulting from maternal uniparental disomy 15 (UPD), and the remainder of cases caused by imprinting center defects (3–5 %) or chromosomal translocations (< 1 %) [4].

Infants with PWS exhibit hypotonia, decreased movement and feeding difficulties [1–3]. Additional clinical manifestations develop in childhood, including short stature, distinct craniofacial characteristics, hypogonadotropic hypogonadism, scoliosis, cognitive delays, behavioral challenges, hyperphagia, and obesity [1–4]. To date, the only approved treatment for PWS is growth hormone (GH) therapy, which is approved for use in children with PWS and has been shown to improve height velocity, body composition, motor development, and quality of life [5–10].

Among the craniofacial features of PWS, a variety of eye and visions issues have been reported, including strabismus. The reported prevalence of strabismus in PWS varies from 28 to 95 % [11], but most studies to date have included relatively small numbers of participants. Additional vision deficits have also been documented in PWS with varying frequencies, including myopia, hyperopia, amblyopia, and astigmatism [11]. Considering the few reports on prevalence of vision issues in PWS, and lack of information on corrective vision surgeries, we used data from the Global PWS Registry [12] to perform a cross-sectional prevalence study on vision problems in PWS.

## Methods

This is an observational retrospective study using data collected from the Global PWS Registry. All protocols and surveys were reviewed and approved by the New England Institutional Review Board (NEIRB). Informed consent was obtained from all subjects or, if subjects were under 18, from a parent and/or legal guardian. Surveys are typically completed by parents/caregivers of individuals with PWS, and in some instances, by the individual with PWS themselves [12]. The study population included participants who completed the vision survey between May-2015 to March-2020. Not all questions were completed by all respondents, thus the number of responses per question varies. Descriptive analyses were performed (proportions, means and standard deviations). Cochran-Mantel Haenzel tests and Chi-Squared tests were performed to compare eye condition prevalence by genetic subtype and growth hormone use. Analyses were performed in Microsoft Excel and SAS 9.4 (SAS Institute, Inc., Cary, NC). *P*-values of < 0.05 were considered statistically significant.

## Results

### Participant demographics

The demographic information for the study cohort is provided in Table 1. This includes participants in the Global PWS Registry who completed the Vision survey over a several year period (May-2015 to March-2020). Ninety-three percent of participants have seen an ophthalmologist at some point in their life.

**Table 1:** Demographic Information of Participants with PWS who completed the Global PWS Registry Vision Survey

Characteristics	Study Participants (*n* = 908) n (%)
Sex	
Female	462 (50.9)
Male	446 (49.1)
*Missing*	3 (0.3)
Mean age at survey completion (years), ±SD	14.5 ± 11.5
Age range (years)	0–62
Genetic Subtype	
Deletion	422 (49.3)
Uniparental Disomy (UPD)	296 (34.6)
Imprinting Defect (ID)	25 (2.9)
Translocation	9 (1.1)
Other	15 (1.8)
Don’t Know	89 (10.4)
*Missing*	52 (5.7)
Country of Residence	
United States	744 (81.9)
Canada	77 (8.5)
Australia/New Zealand	40 (4.4)
Other (24 additional countries)	46 (5.1)
*Missing*	1 (0.1)

*PWS* Prader-Willi Syndrome

a

### Strabismus

**Fig. 1:**
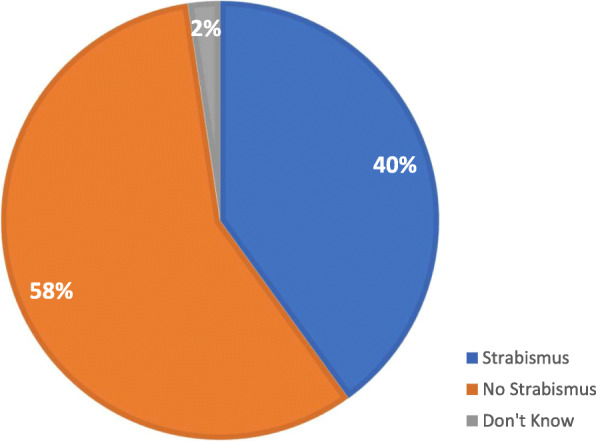
Prevalence of Strabismus among Global PWS Registry Vision Survey. Respondents (*N* = 887) were asked if the participant (the individual with PWS) had “Strabismus (deviated eye, cross-eyed)”

**Fig. 2:**
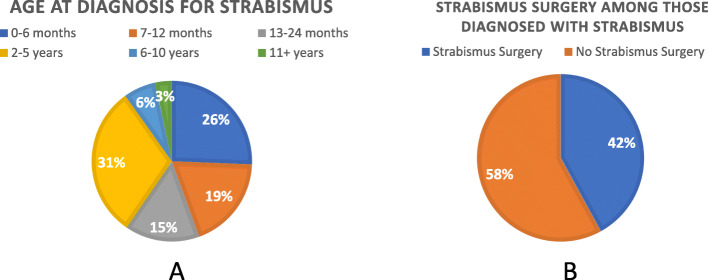
Age at Strabismus Diagnosis and Prevalence of Strabismus Surgery. a**.** For those with strabismus, the age at which survey respondents were diagnosed with strabismus (*N* = 324). **b**: For those with strabismus, prevalence of those who went on to have strabismus surgery (*N* = 139)

**Fig. 3:**
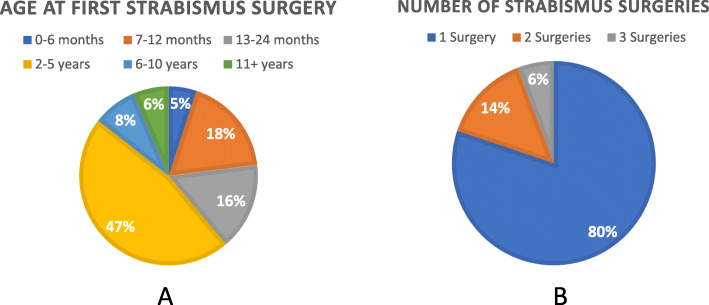
Age at First Strabismus Surgery and Number of Strabismus Surgeries. **a**: For those who ever had strabismus surgery, age at their first strabismus surgery (*N* = 139), and **b**: Number of strabismus surgeries for those who ever had strabismus surgery and reported the number of surgeries (*N* = 139)

The prevalence of strabismus in this study cohort was 40 % (Fig. 1). There was no statistically significant difference in strabismus prevalence between the major genetic subtypes (Deletion: 41.9 %, UPD: 43.6 %, *p* = 0.66), nor by GH use (Never on GH: 41.2 %, GH initiated before 2 years of age: 47.4 %, GH initiated after 2 years of age: 40.3 %, *p* = 0.24). The age at strabismus diagnosis among Registry participants is shown in Fig. 2a. The majority of participants (91 %) were diagnosed before the age of 5. Among the participants who were diagnosed with strabismus, 42 % went on to have strabismus surgery (Fig. 2b). The age of participants at the time of their first strabismus surgery is shown in Fig. 3a. Eighty-six percent of participants had surgery before 5 years old. Among the population who had strabismus surgery, 125 participants had one surgery, 11 participants had two surgeries, and 3 participants had three strabismus surgeries (Fig. 3b).

### Other eye conditions

**Fig. 4:**
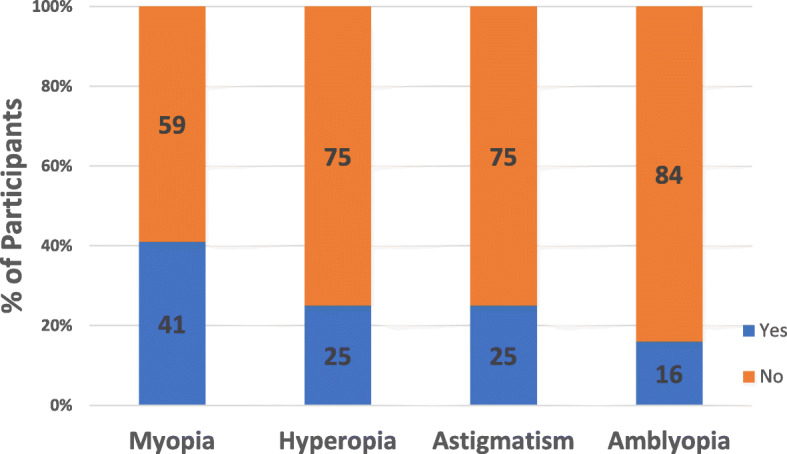
Prevalence of other eye problems in PWS from Vision Survey. Answer options included “Near sightedness, difficulty seeing distant objects”, reported here as myopia, “Far sightedness, difficulty seeing close objects”, reported here as hyperopia, “Astigmatism, curvature of the lens”, and “Amblyopia, lazy eye”. Respondents (*N* = 887) may have received more than one diagnosis of additional eye problems

Additional common vision issues in PWS were also reported in the Vision survey. Forty-one percent of participants had myopia (nearsightedness), 25 % had hyperopia (farsightedness), 25 % had astigmatism (imperfections in the eye curvature), and 16 % had amblyopia (Fig. 4). The prevalence of hyperopia and amblyopia were not significantly different by the major genetic subtypes (Deletion: 26.9 %, UPD: 21.5 %, *p* = 0.11, and Deletion: 17.4 %, UPD: 16.5 %, *p* = 0.77, respectively). However, the prevalence of myopia and astigmatism were significantly different by major genetic subtype. For myopia, the prevalence was higher among those with deletion compared to UPD (44.3 % vs. 35.6 %, *p* = 0.021). For astigmatism, the prevalence was also higher among those with deletion compared to UPD (26.9 % vs. 19.1 %, *p* = 0.018). There was no statistically significant difference in prevalence of other eye conditions by growth hormone use (hyperopia: Ever on GH: 23.7 %, Never on GH: 27.1 % (*p* = 0.51); myopia: Ever on GH: 39.2 % Never on GH: 49.4 % (*p* = 0.07); astigmatism: Ever on GH: 23.7 %, Never on GH: 29.4 % (*p* = 0.26); and amblyopia: Ever on GH: 15.8 %, Never on GH: 17.6 % (*p* = 0.66)). A few other eye conditions were reported (e.g., nystagmus, blocked tear ducts, premature retinopathy), but none in more than 0.33 % of the population.

## Discussion

Although it is generally known that vision issues are common in individuals with PWS, there are few reports in the literature on prevalence, and an absence of information regarding corrective vision surgeries. This report on data from a cohort of participants within the Global PWS Registry highlights the breadth of vision issues in PWS, as well as the prevalence of corrective strabismus surgery.

Among participants within the Global PWS Registry who have completed the vision survey, the prevalence of strabismus (40 %) is much higher than that in the general pediatric population, which ranges between 2.1 and 3.6 %, depending on race and ethnicity [13–15], and also much higher than the prevalence of strabismus among all age groups in the IRIS registry, which contains over 30 million patients (2.75 %) [16]. Previous reports of strabismus among individuals with PWS range from 28 to 95 % [11]. Most recently, a clinical study of the effect of growth hormone therapy on 355 PWS participants reported a strabismus prevalence of 42 %, which is line with the results reported here [17]. That study also showed a statistically higher prevalence of strabismus in individual with PWS by UPD versus those with the deletion subtype (53 % vs. 39 %) [17]. The current study of data from the Global PWS Registry, with almost 3 times the number of subjects, did not confirm this suggested difference in prevalence of strabismus by genetic subtype.

There are several hypotheses for the high rates of strabismus and other vision problems in PWS, including as a consequence of hypotonia, or alterations in typical facial morphology [11]. A 2019 study suggests a positive impact of GH therapy on strabismus, wherein the prevalence of strabismus was significantly lower in PWS individuals who had received GH therapy [18]. However, that study included a relatively small cohort of patients (*N* = 64), whereas a significant impact of GH therapy on strabismus was not reported in a larger PWS natural history study [17], nor in the Registry data represented here. Thus, additional data is needed to understand the impact of GH therapy on strabismus incidence and severity in PWS.

For Registry participants who report strabismus, the vast majority are diagnosed under the age of 5 (91 %). The rate of surgery reported here among PWS individuals with strabismus (42 %) is consistent with a prior study where the percentage was 36 % [19]. Both of these rates are much higher than in the general population where only 5 % of people with a strabismus diagnosis received strabismus surgery [16]. In this report, for those participants who received a corrective strabismus surgery, the majority received their first surgery under the age of 5. This highlights that strabismus in PWS starts at an early age and examination by an ophthalmologist should be a pediatric priority in the PWS population. Importantly, 10.1 % of Registry participants who underwent strabismus surgery went on to experience additional strabismus reoperation in the future, a similar rate to that reported in the IRIS registry (6.72 % reoperation within 1 year of strabismus surgery) [16].

Additional vision issues captured in the Vision survey of the Global PWS Registry include myopia, hyperopia, astigmatism, and amblyopia. Of these, the incidences of hyperopia and amblyopia were higher in this cohort than reported in the general population. Conversely, the prevalence of myopia was comparable to the general population, and astigmatism was lower. Specifically, the prevalence of hyperopia among Registry participants was higher (25 %) compared to the rates found in a meta-analysis of school-aged children (8.4 % among those 6 years old, 3 % among ages 9–14, and 1 % among those 15 years old) [20], and higher compared to adults over 20 in an NHANES survey (3.6 %) [21]. The prevalence of myopia among Registry participants (overall: 41 %; Deletion: 44.3 %, UPD: 35.6 %) was relatively similar to both a large pediatric cohort in California (42 %) [22] and to adults over 20 in an NHANES survey (33.1 %) [21]. Interestingly, the prevalence of astigmatism (overall: 25 %; Deletion: 26.9 %, UPD: 19.1 %) among Registry participants was lower than the 41 % previously reported in individuals with PWS [19] and lower than the prevalence among adults over 20 in a national NHANES survey (36.2 %) [21].

The prevalence of amblyopia among Registry participants (16 %) was higher than the amblyopia prevalence in the general pediatric population, which is reported to range between 1.5 and 2.6 %, depending on race and ethnicity [13, 14]. This is an important issue for follow up because amblyopia is typically considered an avoidable complication if strabismus is diagnosed early and if effective treatment is provided. It raises questions about the utilization and efficacy of patching in this population, and whether PWS alone predisposes or increases the risk of amblyopia.

The high prevalence of a myriad of eye and vision problems in PWS may have far-reaching consequences. For a population that struggles with obesity due to a combination of hyperphagia (food-seeking behavior), hypotonia (poor muscle tone), and a lower resting metabolic rate, any additional impairments or challenges to physical activity can further contribute to weight gain. Impaired eye coordination or vision can negatively impact simple activities like walking, balance, and exercise. Moreover, impaired vision can impact learning, presenting additional challenges to a population that already faces intellectual disability, as well as cognitive and developmental delays.

The data from the Global PWS Registry may be limited by the self-reported nature of the surveys, however, the fact that the findings from this patient reported outcomes method correlate well with other, more traditional, sources, suggests that this is a generally robust mechanism for collecting this type of data on a large scale, complementing traditional natural history studies, which can be prohibitively expensive.

Finally, this report highlights that screening for and correcting vision deficits early in individuals with PWS is an important part of their clinical care.

## Conclusions

This large-scale, representative survey of people with PWS showed a high prevalence of vision and eye problems, including strabismus, myopia, hyperopia, astigmatism, and amblyopia. While the incidence of myopia and astigmatism are similar or lower to what is reported in the general population, the incidence of strabismus, amblyopia, and hyperopia are all considerably higher. Moreover, for PWS individuals with strabismus, multiple corrective strabismus surgeries may be more common than in the general population. Children with PWS should be screened by an ophthalmologist for potential eye and vision issues. Early diagnoses can help families and practitioners develop an effective treatment plan.

## Data Availability

The datasets used and/or analyzed during the current study are available from the corresponding author on reasonable request, and approval from the Global PWS Registry Advisory Board.
